# Cigarette Smoking in Male Patients with Chronic Schizophrenia in a Chinese Population: Prevalence and Relationship to Clinical Phenotypes

**DOI:** 10.1371/journal.pone.0030937

**Published:** 2012-02-07

**Authors:** Xiang Yang Zhang, Jun Liang, Da Chun Chen, Mei Hong Xiu, Jincai He, Wei Cheng, Zhiwei Wu, Fu De Yang, Colin N. Haile, Hongqiang Sun, Lin Lu, Therese A. Kosten, Thomas R. Kosten

**Affiliations:** 1 Menninger Department of Psychiatry and Behavioral Sciences, Baylor College of Medicine, Houston, Texas, United States of America; 2 Beijing HuiLongGuan Hospital, Peking University, Beijing, China; 3 Institute of Mental Health, Peking University, Beijing, China; 4 The First Affiliated Hospital, Wenzhou Medical College, Wenzhou, China; 5 The National Institute on Drug Dependence, Peking University, Beijing, China; Federal University of Rio de Janeiro, Brazil

## Abstract

The high prevalence of smoking in schizophrenia of European background may be related to smoking's reducing clinical symptoms and medication side effects. Because smoking prevalence and its associations with clinical phenotypes are less well characterized in Chinese than European patients with schizophrenia, we assessed these smoking behaviors using clinician-administered questionnaires and the Fagerstrom Test for Nicotine Dependence (FTND) in 776 Chinese male schizophrenia and 560 control subjects. Patients also were rated on the Positive and Negative Symptom Scale (PANSS), the Simpson and Angus Extrapyramidal Symptom Rating Scale (SAES), and the Abnormal Involuntary Movement Scale (AIMS). We found that the schizophrenia patients had a higher lifetime incidence of smoking (79% vs 63%), were more likely to be heavy smokers (61% vs 31%), and had lower smoking cessation rates (4% vs 9%) (all p<0.0001) than controls. Among the schizophrenia patients smoking prevalence increased with age, with the largest difference from controls in the age cohort of 55–75 years: 75% vs 46% (p<0.0001). Among the schizophrenia smokers 73% started to smoke before the onset of their illness by an average of 7.6 years. The patients with schizophrenia who were current smokers scored significantly lower on the PANSS negative symptom subscore (p<0.005), and on the SAES symptom scale (p<0.04; Bonferroni corrected p>0.05) than the non-smoking patients. These results suggest that Chinese males with schizophrenia smoke more frequently than the general population. Further, smokers with schizophrenia may display fewer negative symptoms and possibly less parkinsonism than non-smokers with schizophrenia.

## Introduction

Schizophrenia patients of European ancestry have three to four times higher smoking rates than the general population and other severely mentally ill patients [1]–[4]. Studies show that after correcting for possible confounds, such as marital and socio-economic status, alcohol use, antipsychotic use or institutionalization, higher rates of smoking persist in schizophrenia across cultures and countries [1], [2], [5]. Furthermore, smokers with schizophrenia consume more cigarettes, extract more nicotine per cigarette, prefer brands higher in nicotine, have higher blood nicotine levels, and have lower success rates in attempts to stop smoking than smokers with no mental illness [3], [6]–[8]. High rates of smoking among those with psychotic disorders may contribute to the 20% reduction in life expectancy reported in this population [9]. It is unknown why there is such widespread smoking in patients with schizophrenia. Some evidence seems to support that nicotine may help alleviate negative symptomology, reduce side effects of antipsychotic medications, and possibly ameliorate cognitive deficits [1], [10].

However, evidence is inconsistent regarding the association between smoking and symptoms of schizophrenia [11]. Indeed, in spite of support from animal studies [12], very limited clinical data corroborate an association between smoking and a reduction in negative symptoms [11]. For example, higher levels of both negative and positive symptoms have been found in smoker than in non-smoker schizophrenia, and acute cigarette smoking following 6–12 hours of abstinence reduces schizophrenia' negative symptoms, but several studies find no differences in negative symptoms between smokers and non-smokers [13]–[19]. Others have divided schizophrenia smokers into subgroups based on smoking severity and shown lower levels of negative symptoms and higher levels of positive symptoms in heavy smokers than light smokers [6], [15]. Recently, we found fewer positive symptoms in Chinese schizophrenia smokers than non-smokers, and smoking more cigarettes correlated with fewer negative symptoms [20]. Further studies are needed to clarify the relationship between smoking and symptoms of schizophrenia.

Significantly less Parkinsonism is reported in neuroleptic treated patients with schizophrenia who smoke [16]. This may represent a protective effect of cigarette smoking, since smoking behaviors start decades before the onset of parkinsonism [1], [16]. The association between tardive dyskinesia (TD) and smoking is less clear. Studies show lower rates [21], higher rates [22], [23] and no relationship between smoking and TD [16], [18].

Thus, smoking in schizophrenia may be associated with reduced clinical symptoms and medication-induced extrapyramidal side effects. However, the prevalence of smoking and its associations with demographics and clinical phenotypes have been relatively under-examined among Chinese schizophrenia patients in spite of the large proportion of Chinese who smoke [24], [25], [26]. Gender is a critical demographic determinant of smoking behaviors in all countries, particularly in China, where smoking is extraordinarily rare among females in the general population (male/female: 67.1% *vs.* 7.1%) and in patients with schizophrenia (male/female: 52.0% *vs.* 4.5%) [1], [3], [19], [26], [27]. Because the smoking rate for our schizophrenia female inpatients is also low (5.0%) [20], we focused on male subjects in this study. The purpose of this study therefore was to investigate the smoking prevalence and its associations with clinical symptoms and medication-induced extrapyramidal symptom severity in a large sample of Chinese male patients with schizophrenia.

## Materials and Methods

### Subjects

We approached all inpatients in the Beijing Hui-Long-Guan Hospital, a Beijing City owned psychiatric hospital, and HeBei Province Veteran Psychiatric Hospital in BaoDing city, which is about 50 miles away from Beijing using a cross-sectional naturalistic design. The recruitment criteria included: 1) age 25–75 years, Han Chinese; 2) confirmed DSM-IV diagnosis of schizophrenia; 3) with at least 5 years of illness; 4) had been receiving stable doses of oral antipsychotic drugs for at least 12 months before entry into the study. The mean age of the patients was 47.3±9.8 years. All patients were of the chronic type, with a mean illness course of 22.3±7.2 years. Patients were hospitalized for an average of 8.9±6.9 years. Since these patients were hospitalized, there were restrictions on their smoking, with a fixed schedule for smoking: three or four times each day, and 30 minutes each time. During the smoking period, a patient could smoke as many cigarettes as he/she liked. The patients or their family members had to purchase the cigarettes, with occasional supplemented supplies from their friends or employers, but at very low prices for most cigarette brands. Thus, smoking was not economically limited, and for the assessment peroid of these baseline smoking behaviors no patients were engaged in any behavior reinforcement schedules using cigarettes. The patients in the present study could be considered a representative sample of institutionalized chronic patients with schizophrenia in China.

The resident registration files provided a random sample of control subjects (age 25–75 years) who lived in the Haidian District of Beijing, and we sent each subject a letter explaining the purpose of the study. Of the 812 eligible subjects, 560 completed the baseline interview (participation rate: 69%). Local officials and health centers arranged for the interviews and measurements to take place at the center office at times convenient to the participants. All participants were interviewed by trained investigators supervised by a research psychiatrist. A clinical interview was used to exclude potential controls with Axis I disorders by a research psychiatrist.

We obtained a complete medical history, physical examination and laboratory tests from patients and control subjects. All were in good physical health. Participants were not dependent on alcohol or other substances other than tobacco, based on their self-report. Cannabis use in patients with schizophrenia is extremely rare in China, and our inpatient sample was in a confined setting with monitoring for alcohol and other substances (and any smoked cannabis use would have been particularly easy to detect). All subjects gave signed, informed consent to participate in the study, approved by the Institutional Review Board (IRB), Beijing HuiLongGuan hospital. If a participant had reduced capacity/ability to consent, his/her parents or guardians consented on his or her behalf.

### Measures

Each subject filled out a detailed questionnaire that recorded general information, sociodemographic characteristics, smoking behavior, and medical and psychological conditions. In addition, we administered a cigarette smoking questionnaire to record smoking history and family history of smoking from each subject, including the pack years. The Chinese translation of the standardized Fagerstrom Test for Nicotine Dependence (FTND) was employed to measure the degree of nicotine dependence [28]. Additional information was collected from available medical records and collateral data (from family and/or treating clinician). Additional visits were requested for subjects with missing or ambiguous data.

We divided the subjects into groups based on their smoking history and FTND scores. *Never smokers* were defined as individuals who had smoked less than 100 cigarettes during their lifetime. *Former smokers* were defined as persons who had previously smoked more than one cigarette each day but had quit smoking for more than 1 year. *Current smokers* were defined as persons who smoked more than one cigarette each day and have smoked for more than 1 year. *Ever smokers* included current and former smokers. *Smoking cessation rates* were measured as the ratio of former smokers to the total subjects. *Heavy smokers* were defined as persons who had smoked 15 or more cigarettes per day, or rated with a score of ≥6 in the FTND [29].

### Clinical Measures

Four psychiatrists who had previously completed training in scoring the Positive and Negative Syndrome Scale (PANSS) assessed patient psychopathology using it. After training, repeated assessment showed that they maintained an inter observer correlation coefficient greater than 0.8 for the PANSS total score.

The same clinical psychiatrists assessed medication side effects, Parkinsonism and akathisia using the Simpson and Angus Rating Scale (SAES) and tardive dyskinesia using the Abnormal Involuntary Movement Scale (AIMS).

### Statistical Analysis

Demographic and clinical variables of the smoker and non-smoker groups were compared using *t*-tests and analysis of variance (ANOVA) for continuous variables and chi-squared for categorical variables. Cross-comparisons were performed to calculate smoking prevalence by diagnostic and age group. Odds ratios (OR) derived from logistic regression analyses compared smokers and non-smokers among the patients with schizophrenia and controls after correcting for the related variables. Relationships between clinical variables were examined by calculating Pearson correlation coefficients. Bonferroni corrections were applied to each test to adjust for multiple testing. All p values were 2 tailed with significance level set at 0.05.

## Results

Seven hundred and eighty-nine patients were asked to participate, and 776 consented. However, some subjects did not complete the questionnaire or clinical measures fully; therefore, numbers vary slightly in different categories. The demographic comparisons of the subjects are shown in Table 1.

**Table 1:** Demographics of male patients and male control subjects.

	Schizophrenia(N = 776)	Controls(N = 560)
Age (years)	46.2±9.2	46.7±12.6
Education (years)	9.7±2.5	9.7±3.3
Smoking		
Current smoker	588(75.8%)	302(53.9%)
Former smoker	30(3.9%)	51(9.1%)
Never smoker	158(20.4%)	207(37.0%)

### Comparison of smoking between schizophrenia patients and controls

We compared current smokers with non-smokers (former smokers and those who had never smoked) on demographic and clinical variables for the 776 patients with schizophrenia and the 560 healthy controls in Table 1. As shown, the frequency of ever smoking was 79.6% (618/776) for the patients with schizophrenia and 63.0% (353/560) for the control group, with an OR of 2.29 (95% CI: 2.22–2.36; X^2^ = 45.2, df = 1, p<0.0001). This difference remained significant after using logistic regression to control for the socio-demographic confounds, such as age, education, marital and socio-economic status, and alcohol use (X^2^ = 40.6, p<0.0001, adjusted odds ratio = 2.17; 95% confidence interval, 1.42–2.98).

The frequency of current smoking was 76% for the schizophrenia patients and 54% for the control group, with an OR of 2.67 (95% CI: 2.59–2.74; X^2^ = 69.8, df = 1, p<0.0001). In addition, the frequency of heavy smoking was 61% (476/776) for the patients with schizophrenia and 31% (176/560) for the control group (OR = 3.46; 95% CI: 3.35–3.55; X^2^ = 116.5, df = 1, p<0.0001). The prevalence of smoking cessation was significantly lower in patients with schizophrenia than normal controls [30 of 776 (4%) vs. 51 of 560 (9%); OR = 2.50, 95% CI: 2.24–2.80; X^2^ = 13.8, df = 1, p<0.0001].

### Smoking among the patients with schizophrenia

Of the 618 patients with schizophrenia who had ever smoked, 73% started to smoke before the onset of their schizophrenia, 5% at the onset of illness, and 22% after the onset. The mean age at starting smoking preceded the mean age at onset of illness by 7.6±10.2 years. In addition, there was a positive correlation between age at starting smoking and the age at onset of illness in all patients (r = 0.12, p = 0.005).

### Smokers and non-smokers among the patients with schizophrenia

Smokers were older (mean age = 47.8, SD = 9.2) than non-smokers (mean age = 45.9, SD = 11.4) (t = 2.22, df = 774, p = 0.024), as shown in Table 2. Current smokers also had significantly lower levels of negative symptoms (t = −2.85, df = 762, p<0.005) (Bonferroni correction: p<0.01) and were significantly more likely to be receiving clozapine (X^2^ = 5.29, df = 1, p<0.05). After controlling for the type, the dose (chlorpromazine equivalent) and duration of treatment with antipsychotic drugs, as well as the other related variables such as age, and the number of hospitalizations, the significant difference in negative symptoms between smokers and non-smokers remained (p<0.01). Furthermore, we did not find any significant relationships between smoking and the class, the dose, and duration of treatment with antipsychotic drugs (all p>0.05). Current smokers exhibited less Parkinsonism (p = 0.04), but no difference in tardive dyskinesia on the AIMS compared to non-smokers (p>0.05). However, the Parkinsonism result did not pass the Bonferroni test.

**Table 2:** Characteristics of Smoking and Nonsmoking Patients with schizophrenia.

Item	Smokers(n = 588)	Non-smokers (n = 188)	t or X^2^	df	p
Age (yrs)	47.8±9.2	45.9±11.4	2.22	1,774	0.024
Age at onset (yrs)	23.0±4.7	23.0±4.8	0.09	1,771	0.93
Education (yrs)	8.8±4.5	8.6±2.8	0.44	1,767	0.66
Number of hospitalizations	4.5±2.8	3.6±2.5	2.51	1,760	0.006*
Subtypes of Schizophrenia					
Paranoid type	165 (28.1%)	59 (31.4%)			
Disorganized type	58 (9.9%)	15 (8.0%)			
Residual Type	27 (4.6%)	6 (3.2%)			
Undifferentiated type	321(54.6%)	102 (54.3%)			
Others	17 (2.9%)	6 (3.2%)			
Neuroleptic dose	450.0±442.4	427.6±251.9	0.64	1,767	0.51
(chlorpromazine equivalents, mg/day)					
Antipsychotic types					
Clozapine	288(79.6%)	74(20.4%)			
Non-clozapine	300(72.5%)	114(27.5%)			
PANSS total score	58.8±15.2	61.1±16.9	−1.71	1,762	0.08
P subscore	11.4±4.8	11.2±4.4	0.46	1,762	0.64
N subscore	22.8±7.8	24.7±9.0	−2.85	1,762	0.005*
G subscore	24.6±5.9	25.2±6.8	−1.12	1,762	0.28
Parkinsonism score	1.3±2.1	2.2±2.4	−2.12	1,764	0.04
AIMS total score	4.6±4.4	4.5±4.7	0.31	1,762	0.76
BMI (kg/m2)	24.3±3.7	24.5±4.2	−0.64	1,548	0.52

*indicated the Bonferroni corrected p value, * p<0.05.

## Discussion

We found that among Chinese patients with schizophrenia: (1) current smoking, ever smoking and heavy smoking prevalence was significantly higher than in the general population; (2) smoking cessation rates were significantly lower than in the general population; (3) smokers with schizophrenia had significantly lower PANSS negative symptom scores and tended to have less parkinsonism than non-smokers.

### An association between schizophrenia and smoking behaviors

Our results are consistent with other studies that report high rates of current cigarette smoking and lower smoking cessation rates in patients with schizophrenia than in controls [1], [2]. These associations have been reliably shown in schizophrenia patient cohorts from different demographical areas supporting a possible biological explanation [1]. We also found that 73% of schizophrenia patients started to smoke before the onset of their schizophrenia illness with an average of 7.6 years before illness onset. Thus, pharmacotherapy during schizophrenia or its prodromal period is unlikely to explain the association of this disorder with smoking. The higher frequency of current smoking in patients with schizophrenia appears to result from both a lower quitting rate occurring and a higher and possibly younger initiation rate in this population [1], [30]. As a neurobiological mechanism for this association Freedman et al. [31] have described a genetic neurophysiological abnormality in patients with schizophrenia and their relatives, which nicotine administration temporarily corrects. This abnormality is associated with dysfunctional hippocampal alpha 7 nicotine receptors. Two recent epidemiological surveys showed that new psychotic symptoms were 70% more likely in smokers than nonsmokers in the British general population [32], and that cigarette smoking was associated with greater schizotypy in first degree relatives of patients with schizophrenia [33]. Thus, heritable factors may make subjects who are vulnerable to schizophrenia more prone to become smokers and to start smoking at a young age [1]. Further studies are warranted to investigate this possibility directly.

### Cigarette smoking, symptoms and medication side effects

Two main sub-hypotheses are usually included in the self-medication hypothesis for high smoking rates in schizophrenia: smoking reduces side-effects of antipsychotics; and nicotine may improve schizophrenia symptoms, particularly the negative and cognitive symptoms [15]. Our study supports these hypotheses prediction that schizophrenia smokers would display significantly lower negative symptoms. This finding replicates earlier work in patients with schizophrenia from western regions [13], but is not consistent with others [14], [15], [17]–[19]. Inconsistent findings between studies may be due to differences in experimental procedures, acquiring of data, measurement tests, and parameters.

One mechanism of the self-medication hypothesis is that chronic nicotine can alleviate negative symptoms through dopaminergic effects. Nicotinic acetylcholine receptors have been identified on mesolimbic and nigrostriatal dopaminergic neurons in rats [34], where acute nicotine administration releases dopamine in the striatum and nucleus accumbens [12] and chronic nicotine decreases dopamine catabolism in the dorsal striatum [35]. Since the negative symptoms of schizophrenia are associated with hypoactivity of dopaminergic systems, smoking may lessen negative symptoms by increasing dopamine in the nucleus accumbens [36]. However, positive symptoms are believed to be associated with subcortical dopamine hyperactivity in schizophrenia [36], and we did not find any effects of smoking on positive symptoms. The exact mechanisms for association between smoking and negative or positive symptoms deserve further investigation.

Decreased neuroleptic-induced parkinsonism (NIP), as seen in our schizophrenia smokers replicates some previous studies [16], but not others [17], [23]. A previous study also showed that smoking was associated with lower levels of antipsychotic induced akathisia in schizophrenia [18]. Convincing clinical evidence shows an inverse association between cigarette smoking and lower rates of idiopathic Parkinson's disease (IPD) suggesting a protective effect of smoking [37]. Both IPD and NIP share certain familial, pharmacological, and biochemical characteristics and some patients with NIP may have underlying IPD [38]. Thus, cigarette smoking may prevent, delay the occurrence, or lessen the severity of NIP through effects on IPD risk factors in a patient. Smoking might also have two other mechanisms. First, the direct action of nicotine on enhancing nigrostriatial dopamine release as mentioned above. Second, smoking increases neuroleptic metabolism by inducing hepatic microsomal enzymes [39], which could contribute to lower antipsychotic blood levels and reduced side effects of antipsychotic drugs. Since neuroleptic doses were equivalent for the smokers and non-smokers in our study, cigarette smoking could have improved symptoms of NIP through both enhanced dopaminergic functions and decreased antipsychotic blood levels. Future studies might assess the medication blood levels to distinguish these mechanisms. However, our finding of less neuroleptic-induced parkinsonism in patients who smoked was statistically weak and requires replication.

### Limitations of this study

Several factors limit our findings. First, this is a cross-sectional study design and cannot show direct causality of smoking, whether beneficial or harmful, in patients with schizophrenia. Second, although any axis 1 psychopathology was ruled out in the control group, we did not evaluate any “axis 2” or a “family history of schizophrenia/psychotic illness” in this population. Since Esterberg et al. [33] reported that smoking was more common in schizotypic first degree relatives of patients with schizophrenia, we may have had a small percentage of these persons as controls. Because the community rate of schizophrenia is about 1% and the rate of schizotypy is similarly low, the contribution of this potential confound to overestimating the smoking rate in our controls seems very small. Also, we did not evaluate “subsyndromal psychotic symptoms” in the control population, which is potentially important among younger controls who had not passed through the age of risk for developing schizophrenia. However, we had a relatively older sample (mean 46 years) and did not find that the smoking association was weakened among the older patients and controls. Third, generalizing our study is limited by our sample of chronically hospitalized male patients, who had more severe psychopathology and a longer duration of illness than typical psychotic outpatients or first episode and drug-naïve patients with schizophrenia. Fourth, these patients had a long history of almost 20 years of treatment with antipsychotics. While they had been on their current antipsychotics for at least 12 months (mean treatment 7.5 years), previous antipsychotic treatment, usually with typical rather than atypical antipsychotic treatment may have influenced the effects of smoking to some degree. Hence, examination of a less chronic patient population might show different associations. Fifth, we did not take into consideration a “prodromal phase” or the “duration of untreated psychosis” in determining that the mean age at starting smoking was around 7 years before the onset of clinical illness. The “prodromal phase” is a complex syndrome that is very difficult to date in its onset and length, but seven years appears to be rather long for a “prodromal phase.” However, smoking may be associated with a prodromal period in some patients with schizophrenia. Sixth, our sample was limited to males and can not be applied to females. Seventh, there was no confirmatory measure of smoking in this study, such as expired breath CO, and cotinine levels. Eighth, while smoking may reduce negative symptoms, and possibly NIP in the patients in our present study, the data for medical comorbidity in this group, which could be considerable given their association with smoking, had not been collected. Thus, whether smoking is harmful or beneficial to patients deserves further investigation.

In conclusion, our results extend previous studies linking heavy smoking, greater smoking frequency and lower smoking cessation rates with schizophrenia to a Chinese population. Patients with schizophrenia who smoked displayed significantly fewer negative symptoms and, possibly fewer extrapyramidal side effects lending support for the self-medication hypothesis. However, due to the cross-sectional study design, we cannot attribute causality to our smoking association with schizophrenia. Further studies in first-episode and drug naïve patients with schizophrenia would help clarify the interrelationship between clinical symptoms, antipsychotic-induced side effects and smoking.
